# Prevalence of Acute Symptoms among Workers in Printing Factories

**DOI:** 10.1155/2014/854052

**Published:** 2014-10-16

**Authors:** Somsiri Decharat

**Affiliations:** Department of Industrial Hygiene and Health Science, Faculty of Health and Sports Science, Thaksin University, 222 Papayom District, Phatthalung Province 93110, Thailand

## Abstract

*Objective.* To identify socioeconomic situation factors and behavioral factors associated with the prevalence of acute symptoms among 150 printing workers in 16 printing factories in Southern Thailand. *Materials and Methods.* A cross-sectional study was conducted by interviewing 150 printing workers in 16 printing factories in Southern Thailand. *Results.* Acute symptoms comprised dizziness, drowsiness, eye irritation, light-headedness, rhinitis, shortness of breath, cough, chest tightness, nausea/vomiting, exacerbation of asthma, allergic skin reaction, and visual disorder. The prevalence of symptoms was consistently higher among workers in the printing process than among other workers. Smoking cigarettes and drinking alcohol were not associated with an increased prevalence of acute symptoms among these printing-factory workers. *Conclusion.* The significant associations were found between personal protective equipment and personal hygiene and prevalence of acute symptoms in printing workers.

## 1. Introduction

The printing industry is composed of many different types of business. Almost everything that has print on it has come into contact with some sort of printing business. Thailand's printing industry also uses the best paper, ink, cutters, binders, machinery, and other materials. As a result of ongoing industry support, printing companies and production houses are able to give their customers reliable services and quality printing. Currently, Thailand has >1,208 printing factories, with about 16,200 employees [1]. Each printing process can be divided into 3 major steps: prepress, press, and postpress. Prepress operations include composition and typesetting, graphic arts photography, image assembly, and image carrier preparation. Press refers to the actual printing operation. Postpress primarily involves the assembly of printed materials and consists of binding and finishing operations [2]. Potentially toxic agents occurring in the printing industry include organic solvents, mineral oils, pigments, resins, lead, and paper dust [3]. Workers in this industry have been exposed to these factors through both inhalation and dermal contact. The major printing-factory hazard is exposure to the solvents in inks, thinners, clean-up materials, and so forth [4]. Solvent exposure in offset establishments included white spirits, methylene chloride, isopropanol, 1,1,1-trichloroethane, ethanol, and trichloroethylene [5]. The greatest hazard is exposure through inhalation, causing narcosis (dizziness, headache, nausea, and light-headedness) from acute exposure and skin, liver, kidney, reproductive, and nervous system damage, with repeated exposures. Skin absorption can also produce these effects [6]. The International Agency for Research on Cancer (IARC) classified occupational exposure in the printing industry as possibly carcinogenic to humans and the reported studies did show excesses of lung cancer and bladder cancer and the exposures of interest were considered to include many potential carcinogens [7–9]. Increased risks have also been detected for melanoma [10, 11] and cancers of the buccal cavity and pharynx [12] and pancreas [13]. The US prevalence of asthma in adults by industry of employment, the prevalence, and ORs were significantly elevated for printing, publishing, and allied industries [14]. Several studies have shown a correlation between airborne pollutants and respiratory disorders in which exposure to the latter type of industrial pollutants is associated with a significantly higher prevalence of allergic respiratory diseases [15, 16]. The objectives of this study were to describe the socioeconomic situation(s) of printing workers and worker behaviors and assess the prevalence of acute symptoms among the study cohort of printing workers in the selected printing factories.

## 2. Materials and Methods

This cross-sectional study enrolled 150 workers from 16 printing factories in Southern Thailand with records of higher exposures. The inclusion criteria for the exposed group were printing workers aged 20–60 years, in occupational contact with chemicals (inks, lacquers, adhesives, cleaning solvents, and other chemicals), who had occupational contact with chemicals for at least one year. All 150 workers who were invited to participate in this study agreed to participate.

A standardized, pretested, interviewer-administered questionnaire was used to gather data. The questionnaire collected information on the following variables: general information, work characteristics (e.g., prepress process, printing, and finishing), personal hygiene, and presence or absence of symptoms in the previous 6 months (dependent variables). In addition to factory data, environmental variables included the presence of air ventilation and type (general or local ventilation), wearing a face mask, usually sewing cotton, and working in the factory for >5 years. Sociodemographic variables were gender, age, educational attainment, and monthly income ≥9,000 Baht. Behavioral variables were smoking cigarettes and drinking alcohol. Interviews were conducted after work by trained interviewers. Direct observation was also used to confirm the interview results.

This research was approved by the Ethics Committee of the Institute of Research and Development, Thaksin University. Permission to conduct the study was obtained from the factory owners. All study participants provided signed informed consent.

Data analysis comprised descriptive and analytical components. In the descriptive component, workers' personal and occupational characteristics are presented and their acute symptom rates described. The analytical component assessed the relationships between symptom prevalence and the independent variables described above. All independent and dependent variables were categorical, so chi-square tests were used to assess these relationships. Data analysis was conducted using SPSS for Windows.

## 3. Results

### 3.1. General Characteristics of the Subjects

The descriptive and analytical results are presented below. The distributions for the independent variables are shown in Table 1. From 16 randomly selected printing factories, 150 subjects were interviewed. 53.3% were aged between 20 and 30 years. 41.3% had diploma or equivalent education levels. 64.0% had incomes <9,000 Baht per month. 53.3% of the printing-factory subjects smoked cigarettes and 42.7% drank alcohol.

### 3.2. Work Characteristics of the Subjects

Most subjects (38.7%) worked in the printing process, 31.3% worked in the finishing process, and 30.0% worked in the prepress process. 54.7% of subjects had worked in printing factories for >5 years. Most subjects worked for >8 hours per day and >6 days per week, at 64.0% and 76.7%, respectively. 65.3% of subjects had a local ventilation system (a ventilation hood) in their workplace. Most subjects (86.7%) did not use safety glasses. 58.7% used a cotton mask to protect themselves from factory pollutants that are shown in Table 2.

### 3.3. Prevalence of Acute Symptoms among the Subjects

The prevalence of self-reported symptoms in the past 6 months is shown in Table 3. The prevalence of eye irritation, rhinitis, and allergic skin reaction was >80%. The prevalence of dizziness, visual disorders, and chest tightness was 43.1%, 31.0%, and 27.6%, respectively.

The relationships of positions worked, duration of work, hours worked per day, days worked per week, air ventilation system, and use of PPE independent variables with symptom rates are shown for 3 selected symptoms (eye irritation, rhinitis, and allergic skin reaction) in Tables 4, 5, and 6, respectively. The prevalence of eye irritation was statistically significantly higher among workers in the printing process than among workers in the nonprinting processes. Duration of work, hours worked per day, days worked per week, ventilation system, and mask use were significantly associated with increased eye irritation. Type of ventilation system and wearing safety glasses were significantly associated with reduced eye irritation.

Some independent variables were significantly associated with prevalence of rhinitis. Specifically, prevalence was higher among workers in the printing process and was associated with duration of work, hours worked per day, and days worked per week. Prevalence was significantly associated with wearing a mask at work.

The prevalence of allergic skin reaction was statistically significantly higher among workers in the printing process than other workers. Duration of work, hours worked per day, days worked per week, and mask use were significantly associated with increased allergic skin reaction. Mask use was significantly associated with a lower prevalence of allergic skin reaction.

Statistically significant associations of the independent variables with prevalence of acute symptoms are presented in this study. Male gender and age were not associated with acute prevalence. Significant associations with acute symptoms included duration of work, hours worked per day, days worked per week (years), working in the printing process, air ventilation system, and use of PPE (safety glasses and mask). Working in the printing process was generally positively associated with prevalence (with the exception of dizziness, nausea, and visual disorder). Duration of work, hours worked per day, and days worked per week were also associated with increased prevalence of eye irritation, rhinitis, asthma exacerbation, and allergic skin reaction. Having a local ventilation system and wearing a mask were associated with reduced symptom prevalence. Cigarette smoking and alcohol drinking were not associated with increased symptom prevalence.

## 4. Discussion

The aim of this study was to assess acute symptoms among printing-factory workers. Acute symptoms, including eye irritation, rhinitis, and allergic skin reactions, were significantly more prevalent among workers in the printing process than other workers. This finding was not consistent with Yu et al. [17], who reported that the prevalence of specific symptoms of the nervous system and mucous membrane irritation was significantly associated with low-dose occupational exposure to organic solvents. The finding for allergic skin reaction in this study was similar to Livesley et al. [18], who studied symptoms among printers in the UK printing industry—itching (61%), rash (58%), and dry skin (56%). In addition, a relation was found between working in the printing industry and bronchitis-like symptoms, and although exposure to irritative solvents and paper dust may occur, it is unclear what the main risk factors are within this particular industry (Vermeulen et al. [19]). Nethercott and Nosal [20] found that being an offset lithographic printing operator was related to adverse cutaneous effects, with 67% of operators having allergic contact dermatitis and 29% being caused by ultraviolet (UV) cured ink components. Garabrant [21] assessed the relation between dermatitis and the use of aziridine hardener (TMPTA) used in printing inks and found the incidence to be the highest among ink mixers.

Duration of work, hours worked per day, and days worked per week were statistically significantly associated with prevalence of acute symptoms. This finding was also supported by Vermeulen et al. [19], who focused on respiratory symptoms due to occupational exposures in a contemporary general population cohort. For industries with statistically significant associations with bronchitis and/or asthma symptoms (*P* < 0.10), the regression analyses were repeated based on the total occupational histories with the inclusion of time-related variables, such as duration and time since first employment.

Air ventilation system and PPE use were statistically significantly associated with the prevalence of acute symptoms; this is also consistent with Shusterman [22], who reported that prevention is based on environmental monitoring to control and attenuate exposure to causal agents (substitution, confinement, isolation, and exhaustion), intervention in the organization of work (reduction in the number of environmental stressors, number of persons exposed, and duration of exposure), practice of body and environmental hygiene, periodic medical examinations, training, and use of individual protective equipment (masks, respirators, filters, and air supplies).

Cigarette smoking was not associated with increased prevalence of acute respiratory symptoms; this was inconsistent with studies by Ekburanawat et al. [23], who reported that cigarette smoking was associated with a significantly increased risk of NPC (OR = 2.41, 95% CI 1.61–3.6). Drinking alcohol was not associated with increased prevalence of respiratory symptoms, which was consistent with Ekburanawat et al. [23], who reported that there was no association between alcohol consumption and NPC risk (OR = 0.88, 95% CI 0.58–1.33).

*Limitations to the Present Study.* The study did not ask about any history of common cold or influenza. Colds and flu-like illnesses often involve some of the symptoms assessed in this study. The sample size was limited, with only 150 printing-factory workers being recruited from 16 factories in Southern Thailand. Therefore, the results may not be readily generalizable beyond this sample group in Southern Thailand. Those excluded from the study included workers who were absent from work for whatever reason, those who did not voluntarily participate in the study, and administrative personnel. A multivariate model exceeds the scope of the present analysis.

## 5. Conclusion

In conclusion, chemicals contamination in the workplace should be investigated regularly, with annual health surveillance to evaluate the effectiveness of control measures in the workplace and to protect the health of printing workers. Personal protective equipment, mask, and gloves could help reduce exposure of chemicals in printing workers. In addition, health education would help them to realize the toxicity of chemicals.

## Figures and Tables

**Table 1:** Frequency distributions of general characteristics of the subjects (*n* = 150).

Characteristics	Count (%)
Sociodemographic	
Gender	
Male	98 (65.3)
Female	52 (34.7)
Age (years)	
20–30	80 (53.3)
31–40	48 (32.0)
41–50	22 (14.7)
Education level	
Vocational school	58 (38.7)
Diploma or equivalent	62 (41.3)
Bachelor degree or higher	30 (20.0)
Monthly income (Baht)	
<9000	96 (64.0)
≥9000	54 (36.0)
Behavioral	
Smoke cigarettes	
No	70 (46.7)
Yes	80 (53.3)
Drink alcohol	
No	86 (57.3)
Yes	64 (42.7)

**Table 2:** Work characteristics, work environment (*n* = 150).

Characteristics	Count (%)
Work characteristics	
Position	
Prepress process	45 (30.0)
Printing process	58 (38.7)
Finishing process	47 (31.3)
Duration of work (years)	
≤5	68 (45.3)
>5	82 (54.7)
Hours worked per day	
≤8	54 (36.0)
>8	96 (64.0)
Days worked per week	
≤6	35 (23.3)
>6	115 (76.7)
Environmental	
Air ventilation systems	
General ventilation system (open window and doors)	52 (34.7)
Local ventilation system (hood)	98 (65.3)
Safety glasses at work	
No	130 (86.7)
Yes	20 (13.3)
Cotton mask at work	
No	62 (41.3)
Yes	88 (58.7)

**Table 3:** Prevalence (percent) of acute symptoms during the preceding 6 months (*n* = 150).

Symptom	Count (%)	Worked in printing process (*n* = 58)	Did not work in printing process (*n* = 92)
Dizziness	45 (30.0)	25 (43.1)	20 (21.7)
Drowsiness	32 (21.3)	15 (25.9)	17 (18.4)
Eye irritation	79 (52.7)	55 (94.8)	24 (26.1)
Light-headedness	24 (16.0)	12 (20.7)	12 (13.1)
Rhinitis	75 (50.0)	52 (89.7)	23 (25.0)
Shortness of breath	20 (13.3)	11 (18.9)	9 (9.8)
Cough	24 (16.0)	12 (20.7)	12 (20.7)
Chest tightness	22 (14.7)	16 (27.6)	6 (6.5)
Nausea/vomiting	23 (15.3)	13 (8.7)	10 (6.7)
Asthma exacerbation	26 (17.3)	10 (17.3)	16 (17.4)
Allergic skin reaction	57 (38.0)	48 (82.8)	9 (9.8)
Visual disorder	35 (23.3)	18 (31.0)	17 (18.5)

**Table 4:** Prevalence of eye irritation in the preceding 6 months by independent variable: position worked, duration of work, hours worked per day, days worked per week, air ventilation system, and PPE use.

Independent variable	Prevalence (%)	Count	*P* value
Work position			
Worked in printing process	94.8	55	<0.001
Did not work in printing process	26.1	24	
Duration of work (years)			
<5	15.1	12	<0.001
≥5	84.8	67	
Hours worked per day			
≤8	31.7	25	<0.001
>8	68.36	54	
Days worked per week			
<6	22.8	18	<0.001
≥6	77.2	61	
Air ventilation system			
General system (open window and doors)	69.6	55	<0.001
Local system (hood)	30.4	24	
Used safety glasses at work			
No	74.7	59	<0.001
Yes	25.3	20	
Used mask at work			
No	45.6	36	0.075
Yes	54.4	43	

**Table 5:** Prevalence of rhinitis in the preceding 6 months, by independent variables: position worked, duration of work, hours worked per day, days worked per week, air ventilation system, and use of PPE.

Independent variable	Prevalence (%)	Count	*P* value
Work position			
Worked in printing process	89.7	52	<0.001
Did not work in printing process	25.0	23	
Duration of work (years)			
<5	28.7	29	0.002
≥5	61.3	46	
Hours worked per day			
≤8	25.3	19	<0.001
>8	74.7	56	
Days worked per week			
≤6	30.7	23	0.003
>6	69.3	52	
Air ventilation system			
General system (open window and doors)	50.7	38	0.667
Local system (hood)	49.3	37	
Used safety glasses at work			
No	53.3	40	0.068
Yes	46.7	35	
Used mask at work			
No	73.3	55	0.001
Yes	26.7	20	

**Table 6:** Prevalence of allergic skin reaction in the preceding 6 months, by independent variables: work position, duration of work, hours worked per day, days worked per week, air ventilation system, and use of PPE.

Independent variable	Prevalence (%)	Count	*P* value
Work position			
Worked in printing process	82.8	48	<0.001
Did not work in printing process	9.8	9	
Duration of work (years)			
<5	17.6	10	<0.001
≥5	82.5	47	
Hours worked per day			
≤8	8.8	5	<0.001
>8	91.2	52	
Days worked per week			
≤6	15.8	9	<0.001
>6	84.2	48	
Air ventilation system			
General system (open window and doors)	78.9	45	<0.001
Local system (hood)	21.1	12	
Used safety glasses at work			
No	56.1	32	0.059
Yes	43.9	25	
Used mask at work			
No	64.9	37	0.002
Yes	35.1	20	
